# Advances in the Management of Meconium Aspiration Syndrome

**DOI:** 10.1155/2012/359571

**Published:** 2011-11-22

**Authors:** Kamala Swarnam, Amuchou S. Soraisham, Sindhu Sivanandan

**Affiliations:** ^1^Division of Neonatology, Department of Pediatrics, University of Calgary, Calgary, AB, Canada T2N 1N4; ^2^Alberta Children's Hospital Research Institute for Child and Maternal Health, University of Calgary, Calgary, AB, Canada T2N 4N1; ^3^Department of Pediatrics, Foothills Medical Centre, Rm C211 1403-29th Street NW, Calgary, AB, Canada T2N 2T9

## Abstract

Meconium aspiration syndrome (MAS) is a common cause of severe respiratory distress in term infants, with an associated highly variable morbidity and mortality. MAS results from aspiration of meconium during intrauterine gasping or during the first few breaths. The pathophysiology of MAS is multifactorial and includes acute airway obstruction, surfactant dysfunction or inactivation, chemical pneumonitis with release of vasoconstrictive and inflammatory mediators, and persistent pulmonary hypertension of newborn (PPHN). This disorder can be life threatening, often complicated by respiratory failure, pulmonary air leaks, and PPHN. Approaches to the prevention of MAS have changed over time with collaboration between obstetricians and pediatricians forming the foundations for care. The use of surfactant and inhaled nitric oxide (iNO) has led to the decreased mortality and the need for extracorporeal membrane oxygenation (ECMO) use. In this paper, we review the current understanding of the pathophysiology and management of MAS.

## 1. Introduction

Meconium aspiration syndrome (MAS) is defined as respiratory distress in an infant born through meconium-stained amniotic fluid (MSAF) with characteristic radiological changes and whose symptoms cannot be otherwise explained [1]. Because meconium is rarely found in the amniotic fluid prior to 34 weeks' gestation, MAS is often a disease of the term and near-term infant and is associated with significant respiratory morbidity and mortality. Cleary and Wiswell [2] have proposed a severity criteria to define MAS: (a) mild MAS is a disease that requires less than 40% oxygen for less than 48 hours, (b) moderate MAS is a disease that requires more than 40% oxygen for more than 48 hours with no air leak, and (c) severe MAS is a disease that requires assisted ventilation for more than 48 hours and is often associated with PPHN. In this paper, we look at the current understanding of the pathogenesis and management of MAS.

## 2. Epidemiology of MAS

Meconium is a viscous sticky dark green substance containing gastrointestinal secretions, bile, bile acids, mucus, pancreatic juice, blood, swallowed vernix caseosa, lanugo, and cellular debris. Intrauterine hypoxia may cause passage of meconium in the amniotic fluid. MSAF is present in 8–20% of all deliveries [1–4], increasing to 23–52% after 42 weeks of gestation [5, 6]. Meconium aspiration may occur before birth, or during the birth process. About 2–9% of infants born through MSAF develop MAS [7–9]. About one-third of infants with MAS require intubation and mechanical ventilation [9].

Factors that promote the passage of meconium in utero include placental insufficiency, maternal hypertension, preeclampsia, oligohydramnios, and maternal drug abuse, especially of tobacco and cocaine. The risk of MAS is increased in black Americans, Africans, and Pacific Islanders [7, 10]. Factors associated with the development of MAS among infants with MSAF include thicker consistency of meconium, nonreassuring fetal heart tracing, fetal acidosis, cesarean delivery, meconium below the cords, infants who needed intubation at birth, and a low Apgar score [9, 11]. In the United States, the incidence of MAS decreased nearly fourfold from 5.8% to 1.5% between 1990–1992 and 1997-1998 and this was attributed to a 33% reduction in births at more than 41 weeks' gestation, more frequent diagnosis of nonreassuring fetal heart rate patterns, and greater use of amnioinfusion [12]. MAS remains a serious problem in developing and newly industrialized countries, and MAS accounts for about 10% of all cases of respiratory failure with 39% mortality rate [13].

## 3. Pathophysiology of MAS

MAS results from aspiration of meconium during intrauterine gasping or during the first few breaths. Fetal hypoxic stress can stimulate colonic activity, resulting in the passage of meconium and also stimulates fetal gasping movements that result in meconium aspiration in-utero. Mounting evidence suggests that a chronic in utero insult may be responsible for most cases of severe MAS as opposed to an acute peripartum event [14, 15].

The pathophysiology of MAS is complex. Aspirated meconium can interfere with normal breathing by several mechanisms. The pathophysiologic mechanisms of hypoxemia in MAS include (a) acute airway obstruction, (b) surfactant dysfunction or inactivation, (c) chemical pneumonitis with release of vasoconstrictive and inflammatory mediators, and (d) PPHN with right-to-left extrapulmonary shunting. The common disturbances of lung function in MAS include hypoxemia and decreased lung compliance. Poor oxygenation is attributed to a combination of ventilation perfusion mismatching, intrapulmonary shunting related to regional atelectasis and extrapulmonary shunting related to PPHN.

Depending on the consistency and amount of meconium aspirated, meconium may lead to either partial or complete airway obstruction leading to hyperinflation or atelectasis of the alveoli. The gas trapped may rupture resulting in air leak syndromes such as pulmonary interstitial emphysema, pneumothorax, and pneumomediatinum. 

Presence of meconium in the alveoli can inactivate the endogenous surfactant and decrease the production of surfactant proteins A and B [16, 17]. This causes atelectasis of the lung and can increase ventilation perfusion mismatch. The exact mechanisms for meconium-induced inactivation of pulmonary surfactant are not clearly understood. However, several components of meconium, especially fat-soluble (free fatty acids, cholesterol, and triglycerides), and water-soluble (containing bilirubin, bile acids, enzymes, etc.) ones impair lung function [17]. Meconium can impair pulmonary surfactant by a combined action of cholesterol and bile acid present in meconium [18]. Meconium may also change the viscosity and ultrastructure of the surfactant, decrease the levels of surfactant proteins, and also accelerate the conversion from large, surface active aggregates into small, less active forms. The surfactant dysfunction is enhanced by leakage of plasma protein through an injured alveolar-capillary membrane, as well as the proteolytic enzymes, and oxygen-free radical release from activated cells during the inflammation. 

Meconium can cause chemical pneumonitis. Meconium is a good chemoattractant for neutrophils [19]. Within a few hours, neutrophils and macrophages are found in the alveoli, larger airways, and lung parenchyma. Meconium is also a source of proinflammatory mediators such as interleukins (IL-1, IL 6, and IL 8), tumor necrosis factors. Thus it may induce inflammation either directly or indirectly through the stimulation of oxidative bursts in neutrophils and alveolar macrophages and may injure the lung parenchyma or lead to vascular leakage causing toxic pneumonitis and hemorrhagic pulmonary edema [2].

Acute intrapulmonary meconium contamination induces a concentration-dependent pulmonary hypertensive response, with 15–20% of infants with the MAS showing PPHN. PPHN in infants with MAS may be caused by (a) pulmonary vasoconstriction secondary to hypoxia, hypercarbia, and acidosis, (b) hypertrophy of the postacinar capillaries as a result of chronic intrauterine hypoxia, and (c) pulmonary vasoconstriction as a result of pulmonary inflammation.

Despite the fact that meconium itself has detrimental effects on placental and umbilical tissues in utero, very little is known regarding the meconium-stimulated cellular and biochemical alterations in fluid-filled fetal lungs [20]. However, heavy meconium staining is supposed to inhibit, through unknown mechanisms, fetal lung fluid reabsorption at birth that may disturb the ability of the lungs to adapt properly to extrauterine life [21].

The extent of lung destruction is not closely correlated to the quantity of meconium in lung tissue but rather to the degree of hypoxia and acidosis present at delivery [22]. Ghidini and Spong postulated that severe MAS may not be in fact causally related to the aspiration of meconium but rather caused by other pathologic processes occurring in utero, such as chronic asphyxia, infection, or persistent pulmonary hypertension [15].

## 4. Diagnosis of MAS

It is important to monitor infants born through MSAF for any signs of respiratory distress for at least 24 hours. Diagnosis of MAS is based on the presence of respiratory distress in an infant born through MSAF, with no alternate cause for respiratory distress. Chest radiograph and blood gas analysis should be performed if necessary. Because of diverse mechanisms causing this disease, radiographic findings are different. The classic radiographic findings in MAS are overexpansion of the lungs with widespread coarse, patchy infiltrates. However, the severity of the X-ray pattern does not always correlate with the clinical picture. The lack of correlation between clinical severity and radiographic pattern suggests that MAS is less dependent on the amount of meconium obstruction and parenchymal damage than on other aspects of MAS, such as the presence and severity of PPHN.

## 5. Management of MAS

### 5.1. Prevention of MAS

The decrease in the incidence of MAS in the last decade has been attributed to the reduction in postterm delivery, aggressive management of abnormal heart rate monitoring, and decreased number of infants with a low Apgar score. 

#### 5.1.1. Antepartum Period

Meta-analysis of 14 randomized controlled trials (RCTs) suggests that elective induction of labor for pregnancies at or beyond 41 weeks is associated with significant reduction in the incidence of MAS (RR = 0.43, 95% CI 0.23–0.79) and fewer perinatal deaths (RR = 0.31; 95% CI: 0.11–0.88) compared to expectant management [23].

#### 5.1.2. Intrapartum Fetal Monitoring

Intrapartum monitoring has been recommended to screen for early signs of fetal hypoxia, a risk factor for MAS. There is no evidence that electronic fetal heart rate monitoring (EFM) with or without fetal blood gas and acid-based assessment reduces the risk of fetal or neonatal mortality or morbidity [24]. Fetal scalp pH determination and newer modes like fetal pulse oximetry will improve decision making in timing of delivery and may reduce the incidence of MSAF and MAS [25].

#### 5.1.3. Amnioinfusion

Amnioinfusion has been proposed to reduce the risk of MAS by diluting the meconium, thus reducing its mechanical and inflammatory effects. Amnioinfusion also helps by cushioning the umbilical cord, thus correcting the recurrent umbilical compressions that lead to fetal academia. In a meta-analysis of RCTs, Pierce et al. [26] reported that intrapartum amnioinfusion was significantly associated with reduced risk of MAS (OR 0.30; 95% CI 0.19, 0.46), meconium below the vocal cords, and neonatal acidemia.

In a recent Cochrane meta-analysis of 13 studies, the author stratified the studies based on the clinical settings [27]. Amnioinfusion reduces the risk of MAS only in clinical settings with limited peripartum surveillance (RR 0.25, 95% CI 0.13 to 0.47), but not in clinical settings with standard peripartum surveillance. However, American College of Obstetricians and Gynecologists conclude that routine prophylactic amnioinfusion for the dilution of MSAF is not recommended for the prevention of MAS [28].

#### 5.1.4. Intrapartum Suctioning

In a large multicenter RCT involving 2514 term infants with MSAF comparing intrapartum suction versus no suction, the incidence of MAS (4% versus 4%), mortality, the need of mechanical ventilation, and duration of oxygen therapy were similar in both groups [29]. Therefore, routine intrapartum oropharyngeal and nasopharyngeal suctioning for infants born with clear or meconium-stained amniotic fluid is no longer recommended [30].

#### 5.1.5. Postpartum Endotracheal Suctioning

Neonatal resuscitation program (NRP) recommends intubation and direct endotracheal suction soon after delivery for nonvigorous infants born through MSAF, who have depressed respiratory efforts, poor muscle tone, and/or heart rate less than 100/minute [31]. Cochrane meta-analysis of four randomized studies did not show a difference in the incidence of MAS between intubated and nonintubated vigorous infants [32]. Hence, if the baby born through MSAF has a normal respiratory effort, normal muscle tone, and a heart rate greater than 100 beats per minute, direct endotracheal suction is not recommended. Only suctioning of mouth and nose using a bulb syringe or large bore suction catheter is indicated. According to the International Consensus on Cardiopulmonary Resuscitation and Emergency Cardiovascular Care Science, the available evidence does not support or refute the routine endotracheal suctioning of depressed infants born through MSAF [30].

### 5.2. Treatment of MAS

All infants at risk for MAS who show signs of respiratory distress should be admitted in the neonatal intensive care units. Close monitoring is important since they can deteriorate very quickly. Once the infant develops MAS, management is primarily supportive. Maintenance of optimal thermal environment and minimal handling is essential because these infants are agitated easily, which causes right-to-left shunting, leading to hypoxia and acidosis. Maintenance of adequate oxygenation, optimal blood pressure, correction of acidosis, hypoglycemia and other metabolic disorders is the mainstay of treatment.

#### 5.2.1. Ventilation

Ventilator management of the neonate with MAS is challenging because of the complicated pulmonary pathophysiology resulting from areas of atelectasis and areas of hyperinflation, in association with ventilation-perfusion mismatch and airway compromise [33]. Approximately 40% of babies with MAS require mechanical ventilation and additional 10% require continuous positive airway pressure [34]. There is little evidence from the clinical trials regarding the ventilator treatment of infants with MAS.

Ventilation should be aimed at increasing oxygenation while minimizing the barotrauma that lead to air leak syndromes. The amount of ventilator support depends on severity of respiratory distress. Some infants only require oxygen by hood. In infants with MAS who have hypoxemia (PaO_2_ < 50 mm Hg), hypercarbia (PaCO_2_ > 60 mm Hg), or acidosis (pH less than 7.25) in an oxygen-enriched environment with an inspired oxygen fraction (FiO_2_) > 0.6 are often considered candidates for mechanical ventilation. 

In infants with MAS without associated PPHN, it is sufficient to maintain a pH of 7.3–7.4, with a PaO_2_ targeted between 60 and 80 mmHg and a PaCO_2_ of 40–50 mmHg. Infants may be started with a moderate peak inspiratory pressure (PIP) preferably not exceeding 25 cm H_2_O, a relatively rapid ventilator rate (40–60/min), a moderate positive end expiratory pressure (4–6 cm H_2_O), and an adequate expiratory time (0.5–0.7 sec) to prevent gas trapping and air leaks. If gas trapping is noticed, expiratory time may be increased and PEEP should be decreased (3-4 cm H_2_O) [33].

In infants with MAS and concomitant PPHN, mild hyperventilation and higher FiO_2_ can be considered. But the strategy of achieving hypocapnia and alkalosis by hyperventilation has adverse effects including cerebral vasoconstriction leading to long-term neurologic morbidity as well as air leaks [35, 36]. In such situations other modalities like inhaled nitric oxide and high frequency ventilation should be considered early.

Theoretically High Frequency Ventilation (HFV) minimizes the barotrauma and may reduce air leak syndrome in MAS. No prospective randomized trials have compared conventional ventilation versus HFV in MAS. In pilot studies using inhaled nitric oxide (iNO), Kinsella and Abman [37] found that the combination of HFV and iNO caused the greatest improvement in oxygenation in some patients with severe PPHN. They speculated that improved lung inflation during HFV may augment the response to iNO by decreasing intrapulmonary shunting and improving iNO delivery to the pulmonary circulation [37, 38]. Partial liquid ventilation was found to be a better method of delivering surfactant in an adult rat model of MAS when compared with conventional mechanical ventilation [39]. There is no randomized clinical trial about the use of partial liquid ventilation in human neonates with MAS.

#### 5.2.2. Surfactant Therapy

In vitro studies have shown that meconium interferes with surfactant in several ways: inactivation of its function depending on the concentration, direct toxicity on type II pneumocytes, displacement of surfactant from the alveolar surface, and decrease of surfactant protein A and B levels [2]. 

Canadian Pediatric Society position statement recommends that intubated infants with MAS requiring more than 50% oxygen should receive exogenous surfactant therapy [40]. Surfactant can be given as either a bolus therapy or bronchoalveolar lavage. Bolus surfactant therapy for MAS has been associated with reduction in the severity of respiratory distress and decrease in the number of infants with progressive respiratory failure requiring ECMO. Meta-analysis of 4 RCTs showed reduction in the severity of respiratory illness and decrease in the number of infants with progressive respiratory failure requiring ECMO (RR 0.64, 95% CI 0.46–0.91) [41]. However, there was no significant difference in mortality, hospital stay, length of ventilation, duration of oxygen use, pneumothorax, pulmonary interstitial emphysema, or chronic lung disease. 

Clinical trial of surfactant lavage using Lucinactant in conventionally ventilated infants with MAS found no difference between lavage infants and controls in terms of ECMO requirements, air leak, or duration of ventilation [42]. Similarly, Dargaville and colleagues reported that lung lavage with dilute surfactant (Survanta) in ventilated infants with severe MAS does not decrease the duration of respiratory support, but may produce a reduction in mortality, especially in units not offering ECMO [43].

#### 5.2.3. Role of Steroids

In 2003, Cochrane meta-analysis of two trials [44, 45] including 85 infants with MAS showed that there was no difference in mortality but a small increase in the duration of oxygen treatment in steroid-treated group [46]. Since then, two more trials reported that steroid therapy in MAS was associated with a decrease in the duration of oxygen therapy and hospital stay [47, 48]. The choice of steroid and duration of therapy was different between the studies. Steroids may be beneficial in severe MAS with apparent lung edema, pulmonary vasoconstriction, and inflammation. At present, there is no conclusive evidence to propose routine steroid therapy in the management of MAS. Further research is needed regarding the dosing, timing, and ways of administration of steroids considering their individual properties and possible acute and long-term side effects [49].

#### 5.2.4. Role of Antibiotics

The presence of meconium increases the chances of positive cultures from amniotic fluid in preterm and term infants. However, studies evaluating the development of sepsis in infants with MSAF failed to demonstrate that relationship [50]. Three randomized control studies reported that routine antibiotic prophylaxis is not recommended in the management of MAS for those without perinatal risk factors [51–53]. Antibiotic therapy did not affect the clinical course and outcome related to infection in MAS without perinatal risk factors for infection and without ventilator use. The role of antibiotics in the management of MAS may need to be reevaluated in well-designed trials. Unless there is definite risk for infection, prophylactic use of antibiotics in MAS did not reduce infection. If antibiotics are started for suspected infection due to perinatal risk factors, consider discontinuing antibiotics once the blood culture results are negative.

#### 5.2.5. Nitric Oxide

Severe MAS is often associated with PPHN, resulting in severe hypoxemia. Randomized clinical trials have demonstrated that iNO therapy decreases the need for ECMO in addition to mortality in full-term and near-term neonates with hypoxic respiratory failure and PPHN [54]. For hypoxic respiratory failure due to MAS, infants responded well to combined iNO and HFV as compared to either treatment alone [55]. The response to combined treatment with HFV and iNO reflects both decreased intrapulmonary shunt and augmented nitric oxide delivery to its site of action.

#### 5.2.6. Extracorporeal Membrane Oxygenation

ECMO has been used as a final rescue therapy in infants with severe and refractory hypoxemia associated with MAS. Use of ECMO has been decreased significantly in developed countries with the availability of iNO and HFV. Infants with MAS make up approximately 35% of the infant population who require ECMO [56]. The survival rate has approached 95% of infants with MAS who underwent ECMO [57]. In the ECMO registry, the highest survival rates (>90%) were seen in the patients with MAS who qualified for ECMO [58].

#### 5.2.7. Adjunctive Therapies

All infants with MAS should be monitored using noninvasive monitors (pulse oximeter, transcutaneous O_2_/CO_2_ methods) and blood gas sampling should preferably be done with an indwelling arterial line. Sedation and analgesia are used frequently in infants with MAS and PPHN to alleviate pain and discomfort that may lead to hypoxia and right-to-left shunting. Opioids, particularly morphine or fentanyl, are frequently used to optimize gas exchange and also to avoid asynchrony, reflex catecholamine release, and aggravation of pulmonary vascular resistance.

Depolarizing muscle relaxants (pancuronium, vecuronium) were widely used in the past along with opioids to decrease agitation and subsequent hypoxic episodes in ventilated infants. The benefits of neuromuscular blockade include improved oxygenation, decreased oxygen consumption, and decreased accidental extubations. However, the use of neuromuscular blockade remains controversial and is reserved for the infant who cannot be treated with sedatives alone. Neuromuscular blockage can promote atelectasis of dependent lung regions and ventilation perfusion mismatch and may also be associated with increased risk of death [59]. 

Nearly 30–50% of infants with PPHN do not respond to iNO therapy. Infants who do not show initial response to iNO and those that deteriorate subsequently while on iNO therapy continue to have significant PPHN and need other alternative therapy [60]. Alternatives available include (a) phosphodiesterase-5 inhibitors like Sildenafil, Zaprinast, Milrinone, dipyridamole, (b) prostaglandins like Prostacyclin or PGE1, (c) tolazoline, Magnesium sulfate, (d) NO precursor L-Arginine, (e) free radical scavengers like Superoxide dismutase, (f) experimental agents like Bosentan (endothelin antagonist).

#### 5.2.8. Potential Future Therapy

Currently MAS treatments are all supportive in nature and do not directly affect the injurious actions of meconium on the lung. There is still no effective and safe treatment or prophylactic measure for MAS once the meconium has passed below the vocal cords into the lungs. It has been suggested that fetal pancreatic digestive enzymes play an important role in the lung damage after meconium aspiration by causing disruption of intercellular connections and cell detachment from the basement membrane. A protease inhibitor cocktail prevented the cell detachment induced by meconium suggesting that they may be useful in the treatment and/or prophylaxis [61]. Recent data show that some of the cell death induced by meconium occurs by apoptosis, and therefore has the potential for pharmacologic inhibition through the use of apoptosis blockers or other strategies [62].

## 6. Conclusions

Despite improvement in obstetrical and neonatal care, MAS continues to be a neonatal disorder with high morbidity and mortality. The lung injury caused by meconium is complex and can be attributed to mechanical obstruction of airways, surfactant inactivation, chemical pneumonitis, and PPHN. Among preventive strategies, elective induction of labor for pregnancies at or beyond 41 weeks is associated with significant reduction in the incidence of MAS and amnioinfusion reduces the risk of MAS only in clinical settings with limited peripartum surveillance. Intrapartum management includes endotracheal suctioning to clear meconium only in nonvigorous infants born through MSAF. The management of a symptomatic infant with MAS is primarily supportive. These infants are at high risk of developing PPHN and air leaks. Invasive ventilation if required should use lower PIP, moderate PEEP, higher rates (40–60/min), and adequate expiratory time and permissive hypercapnea should be tolerated to facilitate gentle ventilation. MAS complicated with PPHN and not responsive to conventional ventilation may require HFV and iNO. iNO therapy has decreased the need for ECMO in MAS complicated by hypoxic respiratory failure and PPHN. Surfactant replacement should be considered in ventilated infants requiring more than 50% FiO_2_. Unless there is definite risk for infection, prophylactic use of antibiotics in MAS does not reduce infection or alter the clinical course of illness. ECMO has been used as a final rescue therapy in infants with severe and refractory hypoxemia associated with MAS. The role of steroids and other adjuvant pharmacotherapies like magnesium sulfate, free radical scavengers, and protease inhibitors is still experimental and they are not routinely recommended. As MAS is a major cause of mortality in developing countries, studies focusing on prevention and early treatment should be continued to reduce mortality and morbidity.
